# A Systematic Review and Meta-Analysis of Antibiotic-Impregnated Bone Cement Use in Primary Total Hip or Knee Arthroplasty

**DOI:** 10.1371/journal.pone.0082745

**Published:** 2013-12-12

**Authors:** Jiaxing Wang, Chen Zhu, Tao Cheng, Xiaochun Peng, Wen Zhang, Hui Qin, Xianlong Zhang

**Affiliations:** 1 Department of Orthopaedic Surgery, Shanghai Sixth People’s Hospital, Shanghai Jiao Tong University School of Medicine, Shanghai, China; 2 Department of Orthopaedic Surgery, Anhui Provincial Hospital of Anhui Medical University, Hefei, China; University of Rochester, United States of America

## Abstract

**Background:**

Antibiotic-impregnated bone cement (AIBC) has been widely used for the treatment of infected revision arthroplasty, but its routine use in primary total joint arthroplasty (TJA) remains considerably controversial. With this meta-analysis of published randomized controlled trials, we intended to assess the antimicrobial efficacy and safety of AIBC for its prophylactic use in primary TJA.

**Methods:**

A literature search was performed in MEDLINE, Embase, CBMdisc and the Cochrane Library until June, 2013. The studies were divided into two sub-groups according to the type of the control group. Outcomes of interest included postoperative infection rates, radiographic outcomes and clinical joint score. Study quality was evaluated using the Jadad scale (five points).

**Results:**

In total, eight studies were included, with a sample size of 6,381 arthroplasties. The overall pooled data demonstrated that, compared with the control (plain cement or systemic antibiotic), AIBC did not reveal an advantage in decreasing the rate of superficial infection (relative risk [RR] = 1.47; 95% CI, 1.13–1.91; *P*=0.004), while there were significant differences in deep infection rate between the AIBC and control group (RR = 0.41; 95% CI, 0.17–0.97; *P*=0.04). For the analysis of gentamicin and cefuroxime subgroups, the gentamicin was superior to the cefuroxime in reducing deep infection rate (*P*=0.0005 versus *P*= 0.10). However, no significant differences were found in their radiographic outcomes and clinical joint score.

**Conclusion:**

This meta-analysis had proven that the prophylactic use of AIBC could lower the deep infection rate in primary TJA, while AIBC did not show an improvement in reducing the superficial infection rate compared with the control. More sufficiently powered studies would be required to further evaluate the efficacy and safety of AIBC for primary TJA.

## Introduction

 The use of prophylactic antibiotics and improvements in the operating room environment have helped to reduce the incidence of infection to less than 1% after primary total hip replacement [1] and to 3% after primary total knee replacement [2]. However, surgical site infection following total joint arthroplasty (TJA), especially deep-wound infection, is still a serious complication which can require costly revision surgery, reduce patient’s functional condition and result in prolonged hospitalization. Systemic antibiotics, which are commonly used to prevent or treat periprosthetic infection associated with arthroplasty, are not adequately effective to eradicate deep infection because of the impaired blood circulation and low antibiotic concentrations at the implantation site [3]. Additionally, high dosage of parenteral antibiotics can cause systemic toxicity and other side effects. 

 With the risk of systemic toxicity reduced and sufficient antibiotic concentrations released, local antibiotic delivery is now regarded as an effective method to prevent or treat deep infection following TJA, and among current vehicles used for local drug delivery, antibiotic-impregnated bone cement (AIBC) has become the most frequently used standard vehicle [4]. Since AIBC was introduced by Buchholz and Engelbrecht in 1970 [5], as antibiotic-impregnated cement spacers or beads, it has become more commonly used for treatment of established infection in revision TJA than it has for infection prophylaxis in primary TJA. Since 2003, the United States Food and Drug Administration (FDA) has approved the use of AIBC for second-stage re-implantation after revision due to infection with fixing precise doses, but its use in primary TJA represents an off-label usage [6]. Now, in some European countries, the application of AIBC in primary TJA has also been a standard and common practice for many years, such as Sweden and Norway, however, its use in other European countries is still a matter of debate [6-8]. 

 Despite AIBC having the advantage of reducing the risk of deep periprosthetic infection, there have also been some worries regarding the addition of antibiotics to bone cement and its routine use in primary TJA. The main disadvantages are the possible development of antibiotic resistance, allergic reaction, toxicity, and possible compromise of the mechanical properties of bone cement, and increased cost [9-11]. In 2008, one similar meta-analysis on this topic performed by Parvizi et al. [12] reported a reduction in infection and revision rates for primary total hip replacement (THR) when AIBC was used. Nevertheless, this paper included some nonrandomized studies and their results should thus be treated with caution. Hence, we conducted an updated systematic review and meta-analysis to determine the effectiveness of AIBC use during primary TJA in reducing the rate of surgical site infection, including superficial and deep infection. A secondary aim was to evaluate whether impregnating cement with antibiotic had adverse effects on the survivorship of primary TJA. 

## Materials and Methods

### Study Design

 A meta-analysis and systematic review was conducted according to predefined guidelines provided by the Cochrane Collaboration (2008) [13]. All data were reported according to the Quality of Reporting for Meta-analyses provided by the Handbook for Systematic Reviews of Interventions Version 5.0.0 [14].

### Literature Search

 We performed a literature search to identify all published randomized controlled trials (RCTs) that evaluated the use of AIBC in patients undergoing primary total hip arthroplasty (THA) and/or total knee arthroplasty (TKA). The most common databases included Medline, Embase, CBMdisc, and the Cochrane Library. These were searched for articles published without language and time limitation. The key words used were “hip arthroplasty/ replacement”, “knee arthroplasty/replacement”, “joint arthroplasty/replacement”, “antibiotic cement”, “cement”, and “randomized controlled trial”. In order to collect relevant literature as many as possible, we also used “bone cement”, “antibiotic”, “gentamicin”, “cefuroxime”, “tobramycin”, and “acrylic” as the primary search terms as well as combined with various limiting requirements such as “arthroplasty”, “hip replacement”, and “knee replacement”. The latest date for this search was June, 2013.

### Inclusion and Exclusion Criteria

 In order to be considered eligible for inclusion, studies needed to : (1) include patients undergoing a primary THA or TKA; (2) include an AIBC trial group and a control group that involved the use of plain bone cement (PBC) or systemic antibiotic (SA), irrespective of the dose and route of administration; and (3) be a published RCT. Studies were excluded if: (1) the outcomes were not reported for antibiotic cement use in primary total hip or knee replacement; (2) it was impossible to extrapolate or calculate the necessary data from the published results; (3) primary study patients had a poor physical condition, such as diabetes, malignant tumor; and (4) studies were animal experiments, in vitro trials or revision arthroplasty, and the operated joint was not the hip or knee. The general characteristics, treatment or intervention types, and outcomes were recorded for each study. For studies without the outcomes we need, author(s) would be contacted via e-mail for more relevant information, if necessary. Additionally, it is notable that three separate articles published by Josefsson et al. [8,15,16] described cumulative infection rates in a multicenter trial after three periods of follow-up (2,5 and 10 years). A total of 1688 consecutive primary THAs were randomly treated with either AIBC or SA. There was significantly (*P*<0.05) lower deep infection rate with antibiotic cement after 2 years (0.6% versus [vs.] 1.6%) and 5 years (0.8% vs. 1.9%) of follow-up. Nevertheless, ten years postoperatively, after reclassification of some cases, infection rates were not significantly different between AIBC and SA (1.1% vs. 1.6%). Furthermore, adequate explanation and justification for the reclassification was not provided by the authors, which suggested that these 10-year results should be interpreted cautiously. The data were extracted by 2 reviewers (Xiaochun Peng and Wen Zhang) independently to ensure accuracy. In cases of disagreement, a consensus was reached by discussion and was eventually determined by the senior author (Tao Cheng).

### Methodological Quality Assessment

 The methodological quality of all included trials was graded using the five-point Jadad scale [17]. Any disagreements on study quality were resolved through reviewing the study and discussing the discrepancy. This widely used scale evaluated the reporting of studies based on three fundamental methodological criteria: the method of randomization, adequacy of blinding and the completeness of follow-up. The minimal and maximal scores for an included study were 1 and 5, respectively. We arbitrarily classified quality as high (score: 3-5) versus low (score: 0-2).

### Outcome Measures

 The primary outcome assessed in this analysis was postoperative infection rates between the AIBC and control group, which contained superficial and deep infection rate. The secondary outcome measures included radiographic evaluation (the aseptic loosening) and clinical joint score. A diagnosis of noninfectious loosening required all the following criteria: pain, normal erythrocyte sedimentation rate (ESR), bacteriologic cultures from deep biopsies to be negative and/or a radiolucent zone between the cement and the stem prosthesis [15,18].

### Subgroup Analysis and Investigation of Heterogeneity

Considering whether bone cement was used in the control group, the surgical wound infection (superficial and deep) rate data were divided into 2 subgroups (AIBC vs. PBC and AIBC vs. SA) in each study. If any heterogeneity was observed, the cause of heterogeneity was first analyzed and then subjected to subgroup treatment.

### Statistical Analysis

The data were pooled using REVMAN 5.0 software (The Nordic Cochrane Centre, Copenhagen, Denmark). For each study, we calculated RRs with 95% confidence intervals (CIs) for dichotomous data and mean differences (MDs) with 95% CIs for continuous data. Where appropriate, we pooled the results of comparable groups of trials using the fixed-effect (Mantel-Haenszel test) or random-effect (DerSimonian-Laird method) models, and the model was determined by the total pooled results regardless of the results of each subgroup. A random-effect model was used when significant heterogeneity was detected between studies (*P*<0.10; I^2^>50%). Otherwise, a fixed-effect model was used.

## Results

**Figure 1:**
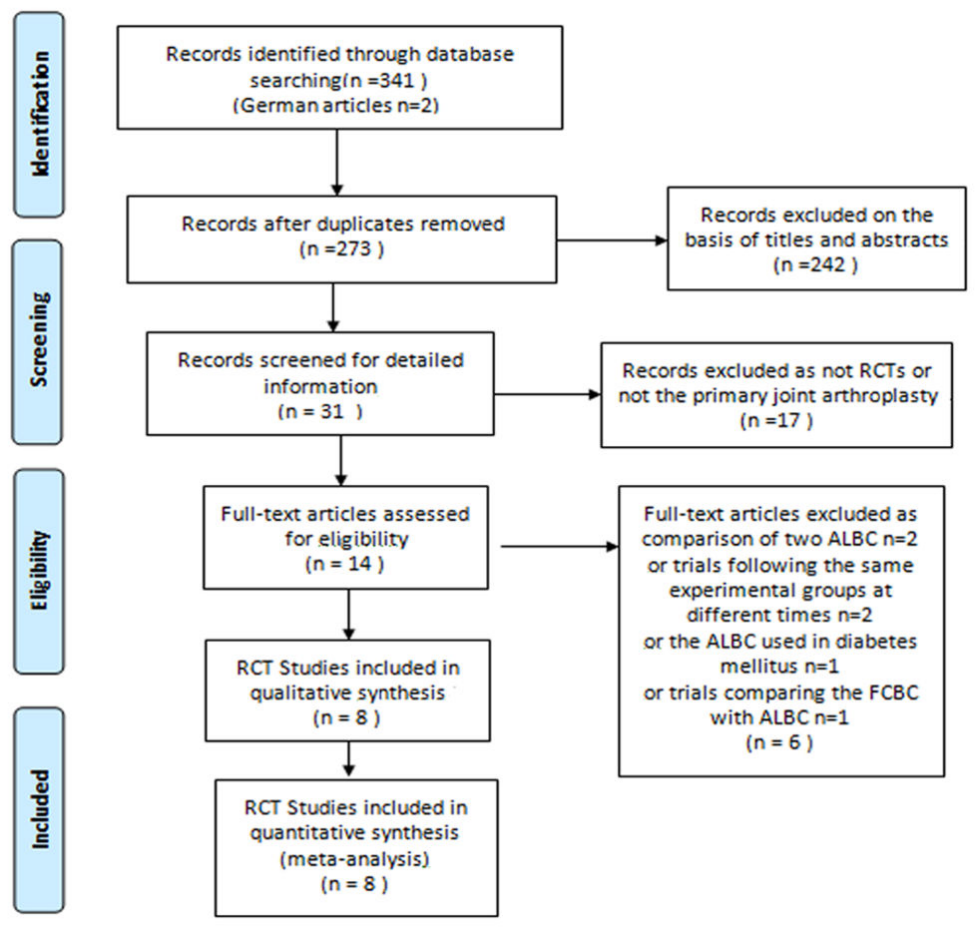
Flow diagram of the selection of studies included in the review. [RCT=randomized controlled trial; FCBC= fluoride-containing bone cement; ALBC=antibiotic-loaded bone cement].

Our search revealed 341 eligible articles, of which 242 were rejected on the basis that the title and abstract were irrelevant. The remaining 31 studies were retrieved for full papers and 17 articles were excluded because these studies did not involve primary TJA or were not RCTs. Subsequently, according to our inclusion and exclusion criteria, we further excluded 6 unsuitable studies. Lastly, the remaining 8 RCTs were included in our meta-analysis. A flow chart detailing the study selection was shown in Figure 1. 

The general characteristics, intervention measures, and outcomes of interest extracted from eight RCTs were displayed in Table S1 in File S1. In total, 6318 arthroplasties were included in our study; 3217 of these arthroplasties received AIBC and 3101 arthroplasties served as the control. Among the eight studies, 6 studies were from European countries, 1 from Canada, 1 from Taiwan. There were 4 studies which reported only THA, 2 only TKA, 2 both THA and TKA. Seven RCT studies provided relevant information about deep infection and the superficial infection only could be extracted from five studies. Four articles referred to radiological accessment, of which one reported a specific evaluation for radiological results. And two articles involved in clinical joint score. All studies reported the type of antibiotic used in cement and five reported the antibiotic dose in cement (per 40 g of bone cement). All papers also showed the type of bone cement, including CMW (DePuy Orthopaedics, Warsaw, IN, USA), Palacos (Zimmer, Inc., Warsaw, IN, USA) or Simplex P (Stryker corporate, Kalamazoo, MI, USA). 

The allocation concealments of six eligible studies were unclear. Only two of these eight reports included adequate blinding procedures. The total length of follow-up was variable, ranging from three months to 49 months. Josefsson et al. [8,15,16]reported three different follow-up periods of 2, 5 and 10 years, and we only chose the minimum to keep the consistency with other included studies in follow-up time. Besides, as the follow-up time increased, we considered that these elderly patients maybe had a worse physical condition, which would have an effect on postoperative complications. More detailed information on the quality of the included RCTs was presented in Table S2 in File S1.

### Post-operative Superficial and Deep Infection Rate

**Figure 2:**
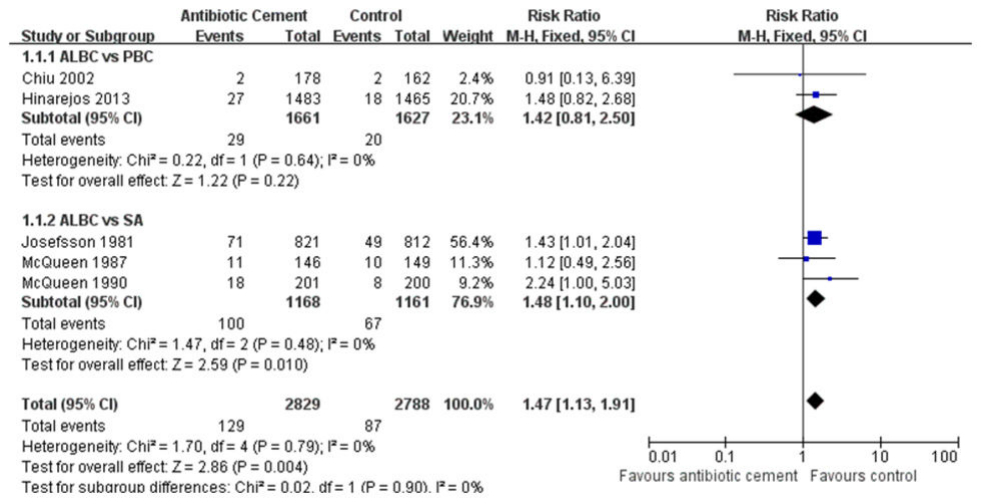
The RRs and 95% CIs for the incidence of superficial infection among patients treated with vs. without antibiotic bone cement. (ALBC vs. PBC and ALBC vs. SA) [ALBC: antibiotic-loaded bone cement; PBC: plain bone cement; SA: systemic antibiotic].

**Figure 3:**
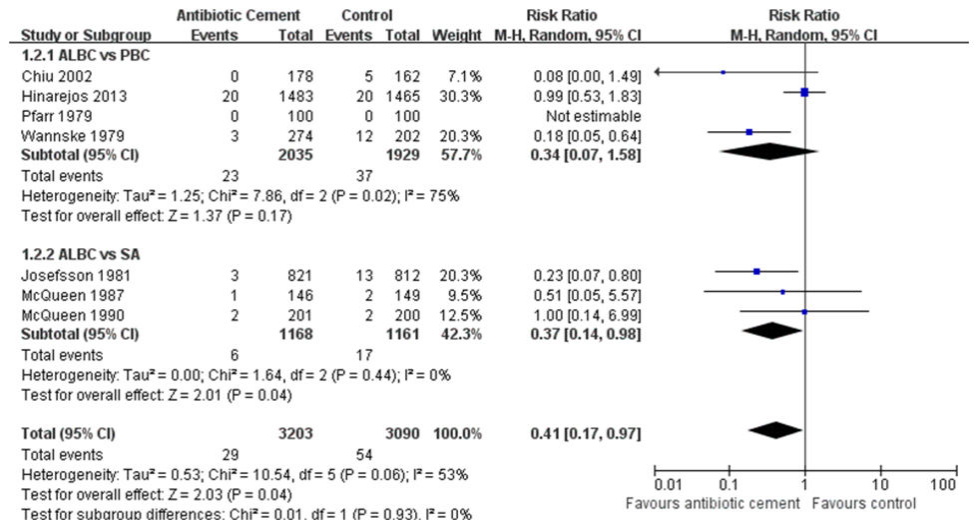
The RRs and 95% CIs for the incidence of deep infection among patients treated with vs. without antibiotic bone cement. (ALBC vs. PBC and ALBC vs. SA) [ALBC: antibiotic-loaded bone cement; PBC: plain bone cement; SA: systemic antibiotic].

**Figure 4:**
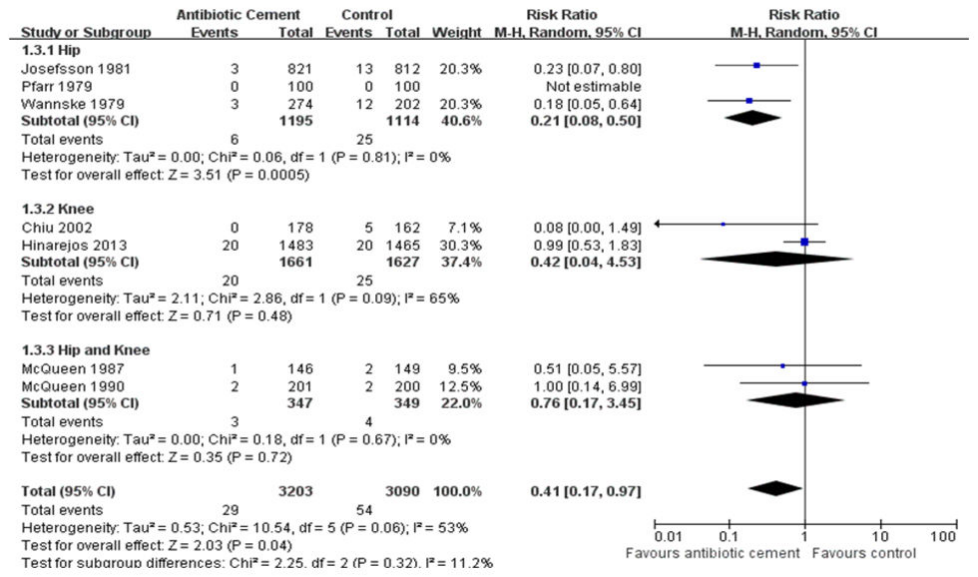
The RRs and 95% CIs for the incidence of deep infection among patients treated with vs. without antibiotic bone cement. (Hip, Knee, Hip and Knee).

**Figure 5:**
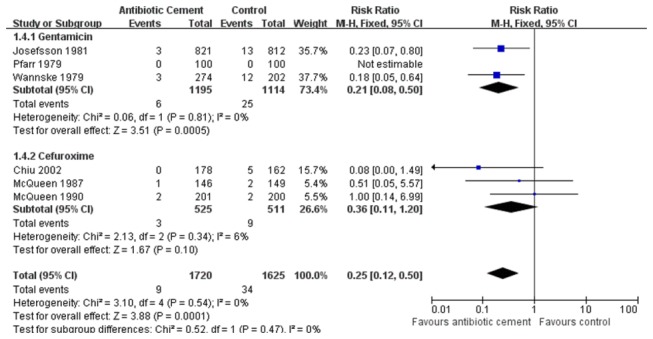
The RRs and 95% CIs for the incidence of deep infection among patients treated with vs. without antibiotic bone cement. (Gentamicin and Cefuroxime).

We included the seven RCTs which involved the postoperative infection rate of patient as the data of the meta-analysis in Table S3 in File S1. In the aspect of superficial infection rate, because no significant heterogeneity was observed among the subgroups (*P*= 0.79; I^2^= 0%), a fixed-effect model was employed. The overall pooled results of 5 RCTs revealed a significant difference between AIBC and control group (RRs, 1.47; 95% CIs, 1.13 to 1.91; *P*= 0.004) (Figure 2). Furthermore, we found different results based on the respective analysis of two subgroups. In the subgroup of AIBC vs. SA, SA had a lower superficial infection rate than AIBC (*P*= 0.01). However, in the subgroup of AIBC vs. PBC, the pooled results showed that there was no statistically significant difference (*P*= 0.22). For deep infection, heterogeneity between the two subgroups was statistically different (*P*= 0.06; I^2^=53%), so we used a random-effect model to evaluate the deep infection rate. The total pooled results exhibited a significant statistical difference between AIBC and control treatments (RRs, 0.41; 95% CIs, 0.17 to 0.97; *P*= 0.04) (Figure 3). In the subgroup of AIBC vs. PBC (*P*= 0.02; I^2^= 75%, a random-effect model), the pooled results showed no statistically significant difference (RRs, 0.34; 95% CIs, 0.07 to 1.58; *P*= 0.17). But in the AIBC vs. SA subgroup (*P*= 0.44; I^2^= 0%, a random-effect model), we found that the deep infection incidence of AIBC was lower than that of SA (RRs, 0.37; 95% CIs, 0.14 to 0.98; *P*= 0.04). In addition, we analyzed the deep infection rate once again among the 3 different subgroups (Hip, Knee, Hip and Knee) based on the targeted joint in each study. With a random-effect model used, the subtotal pooled results also demonstrated the AIBC treatment in THA had a significant advantage over the control group in the prevention of deep-wound infection (*P*=0.0005), while no statistical difference were found between AIBC and control treatments in TKA or THA and TKA subgroups (Figure 4). Moreover, we also observed that among these seven RCTs, two types of antibiotics, namely gentamicin and cefuroxime, were most frequently added in bone cement. With three RCTs included in each subgroup, our overall pooled results showed the significant difference in deep infection rate between AIBC and control patients following primary TJA (P=0.0001, Figure 5). In gentamicin subgroup, we found no significant heterogeneity between studies (*P*= 0.81; I^2^ = 0%) and thus compared the data with a fixed-effect model. The pooled result revealed a significant difference between two treatment groups (RRs, 0.21; 95% CIs, 0.08 to 0.50; *P*=0.0005). In cefuroxime subgroup (P =0.34; I^2^ =6%; a fixed-effect analysis), there was no statistically significant difference between AIBC and control arthroplasties (RRs, 0.36; 95% CIs, 0.11 to 1.20; *P*= 0.10).

### Publication Bias

**Figure 6:**
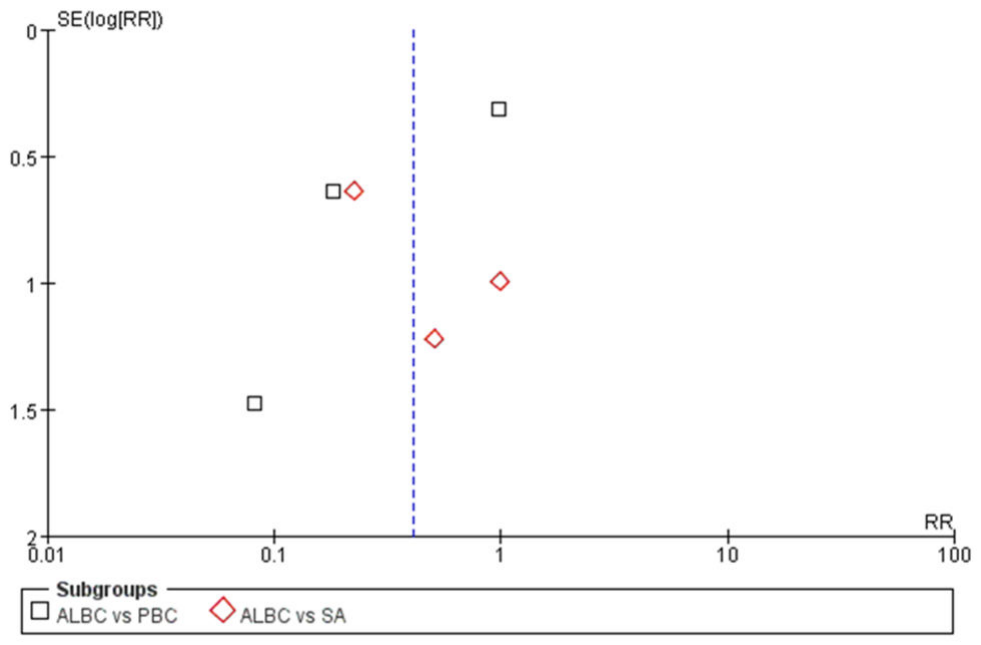
Funnel plot to assess publication. Funnel plot to assess publication for the most frequently reported outcome-the deep infection rate. (ALBC vs. PBC and ALBC vs. SA) [ALBC: antibiotic-loaded bone cement; PBC: plain bone cement; SA: systemic antibiotic].

Publication bias was assessed with funnel plots, which demonstrated the relationship between the study sample size and the precision in estimating the treatment effect. For the analysis of deep infection rate, the funnel plot visually displayed mild asymmetry, suggesting minimal evidence of publication bias (Figure 6).

### Radiographic Assessment

Due to a lack of adequate RCTs, we depicted postoperative aseptic loosening rate based on the data of four published studies during their follow-up time. The trial of Pfarr et al. [19] provided the number of the aseptic loosening joints, but did not show a statistical analysis result. Josefsson et al. [15] reported that the number of aseptic loosening joints in AIBC group was fewer than that in control group (*P*=0.03) and Chiu et al. [20] found that no significant difference existed in aseptic loosening rate between the two groups (Table S4 in File S1). Moreover, the migration of femoral component was analyzed by using radiostereometric analysis (RSA) in another RCT study [21] (Table S5 in File S1). The general conclusion of the study was that no clinically significant differences in stem subsidence or retroversion were found between AIBC and control group during a two-year follow-up period.

### Clinical Joint Score

One study assessed the hip function by Harris hip score (HHS) and one study evaluated the knee function by the Hospital for Special Surgery score. For each study, no statistically significant differences in clinical score were found between AIBC and control group, either preoperatively or the end of the follow-up visits (Table S6 in File S1), which suggested that the admixture of antibiotic in cement would not exert an effect on joint function after primary TJA. And the postoperative joint score was much higher than the preoperative.

## Discussion

Though the addition of antibiotics to polymethylmethacrylate cement with demonstrable elution over a period of time has been shown to be effective in the treatment of established periprosthetic infection [8,15,22], the prophylactic use of antibiotic cement for deep infection after primary total hip or knee replacement remains controversial. The primary finding of our study was that these treatments (AIBC vs. PBC or SA) in primary TJA presented a difference in postoperative superficial infection and deep infection. However, no significant differences were found in their radiographic outcomes and clinical joint score. 

For superficial infection prevention, our results hinted that when SA was used as the reference, the incidence of superficial wound infection was higher in the AIBC group than that in the control group, possibly because antibiotic cement, in contrast with SA, could not supply the superficial parts of the wound with sufficient drug concentrations which could inhibit the growth of bacteria or kill the bacteria. For deep infection intervention, although the use of erythromycin and colistin-loaded bone cement in primary TKA did not lead to a decrease in the rate of infection when SA was used as the control [23] and McQueen et al.[24] found a nearly identical deep infection rate among the last 401 arthroplasties treated with either AIBC or SA in a two-year follow-up report, our comparative analysis had demonstrated a clear benefit of AIBC which led to a statistically significant reduction in the incidence of deep infection following primary TJA. Through the sensitivity analysis, we found that two studies by Josefsson et al.[15] and Hinarejos et al.[23] reported different research results on deep infection rate and the data determined the final analysis results in their respective subgroups. One possible explanation may be that the sample size in both reports was larger than that in other studies. However, with or without these two studies, our overall pooled results both revealed that the AIBC group had a better effect than the control group in the prevention of deep infection (*P*<0.05). Meanwhile, our result on deep infection rate was in line with the result of Parvizi et al.[12]. Compared with their meta-analysis,which had shortcomings in the included literatures and methodology quality, our study, incorporating two different joint replacements (hip and knee), contained only RCTs and obtained more rigorous assessments. In the end, we compared the efficacy of one AIBC with that of another with respect to the type of antibiotic used. For the antibacterial analysis of gentamicin and cefuroxime in preventing deep infection (bone cement from Palacos, Simplex P or CMW), we found that bone cement containing gentamicin was superior to the control group while there was no statistical difference between the cefuroxime-loaded cement and the control group. The possible reasons may be as follows. For one thing, as we know, gentamicin is an ideal antibiotic for inclusion in bone cement as it possesses the characteristics of broad antibacterial spectrum, low protein binding, low sensitisation potential and high water solubility. Simultaneously, gentamicin has unique advantages such as thermal and chemical stability compared with other antibiotics [25]. For another, early sufficient antibiotic concentrations, which are crucial for deep infection prophylaxis, depend on the release of AIBC around the joint prosthesis. Elson et al. [26] and Holm et al. [27] both reported that Palacos cement released higher antibiotic concentrations than CMW, Simplex and Sulfix brands of cement in in vitro studies. In our study, we found that the gentamicin group combined with bone cement Palacos, while cefuroxime group with Simplex P or CMW.

While the finding that antibiotic cement reduces the deep-wound infection rate is promising, there are some concerns on the detrimental effects of adding antibiotics into bone cement. In 2000, Kühn [28] reported that the addition of any substance to bone cement, such as antibiotics, could have an effect on its mechanical properties. Our results did not show higher rates of aseptic loosening in arthroplasties with AIBC than those in the control. Moreover, another randomized trial by Adalberth et al. [29] demonstrated CMW-1 bone cement with gentamicin performed a stable position as well as Palacos R with gentamicin concerning the fixation of the tibial component, compared with PBC, without differences existed in the number, size and extent of radiolucent lines or clinical outcome. Therefore, to some extent, these RCT reports clearly illustrated that the appendage of antibiotics to bone cement would not change its mechanical property regardless of the type of bone cement. Meanwhile, clinical studies showed that low-dose (≤2g of antibiotic powder per 40g cement) AIBC would not lead to an increase in the mechanical loosening rate [30], and high-dose (>4.5 g of gentamicin powder per 40g cement) AIBC or the admixture of liquid antibiotics could cause a decrease in mechanical strength of antibiotic cement [31,32]. It had also been demonstrated that, in comparison with hand-mixing, vacuum-mixing significantly increased the tensile fatigue strength of bone cement (*P*< 0.0001) [33]. For the aseptic loosening of prosthesis, although some risk factors which could affect the mechanical strength of bone cement have been fully clarified above, the last and perhaps greatest underlying cause might be attributable partly to undiagnosed subclinical infection [34,35]. In comparison with PBC or SA, AIBC provided adequate and effective bactericidal or bacteriostatic concentrations over a longer duration by prolonged elution of antibiotics into joint space, and early subclinical infections might be less likely to develop and proliferate. This, in return, provided strong support evidence for the mechanical stability of prosthesis and bone cement. 

So far, no relevant RCT clinical researches could be found to evaluate other adverse issues including antibiotic resistance, allergic reaction, toxicity, and increased cost. However, all of these matters should be paid more attention. Firstly, the emergence of drug-resistant organisms is becoming an ever-increasing societal concern. Several studies had demonstrated the adherence and growth of bacteria on AIBC, which might provide some likely explanations for the emergence of bacterial resistance to antibiotics [36,37]. However, other clinical trials proved that prophylaxis use of AIBC over a short period into a healthy host with low virulence loads did not lead to bacterial resistance [38]. Secondly, to our knowledge, there have been no particular reports of toxicity and allergic reactions related to the use of low-dose AIBC in primary TJA. Now, though the results of in vitro studies raised some concerns that were more relevant with high-dose AIBC (local concentrations of antibiotics exceeding 2000 μg/mL) [39], we did not search any clinical evidence of low-dose AIBC on negative cellular effect (mainly osteoblast and osteocyte). Nevertheless, our surgeons should be cautious to avoid use of a particular antibiotic in bone cement if the patient has a documented allergy to that antibiotic. Thirdly, given that the cost to treat an infected arthroplasty is many times higher than the cost of the initial procedure, we should make a balance between the increased initial cost associated with the use of AIBC and the potential cost savings associated with a realized reduction in deep infection rate in primary TJA. Recently, Cummins et al.[40] employed Markov decision model that accounted for competing risks, benefits, and costs to evaluate the cost-effectiveness of the use of AIBC for primary THA. They thought that when revision due to either infection or aseptic loosening was considered to be the primary outcome, the use of antibiotic resulted in an overall cost decrease.

### Limitations

Although we attempted a well-designed study, some limitations inherent were inevitably found in our meta-analysis. Firstly, there was a paucity of eligible RCTs, especially long-term prospective RCTs, evaluating the role of AIBC in primary TJA. Even though each of the 8 studies claimed to be a RCT, 6 articles did not report their randomization approach with sufficient details to meet CONSORT (Consolidated Standards of Reporting Trials) requirements. This might not necessarily indicate inadequate randomization, but a lack of clarity created uncertainty regarding the strength of the research findings. Of all the included RCTs, only two received a Jadad score>2, and most were rated of fair methodological quality based on the internal validity scale promulgated by the US Preventive Services Task Force. Moreover, due to older vintage, a majority of our included literatures simply did not have the level of methodological details commonly accepted today. Secondly, the original studies were conducted in different hospitals around the world with different patient populations. Different AIBC and a variety of artificial joint prosthesis were used in these studies, which might have affected the outcomes. Different types of bacteria in specific regions would also exert an effect on the incidence of postoperative infection. Thirdly, publication bias associated with funnel plot asymmetry may have affected the results; the present review did not search unpublished studies. In the pertinent trials, incomplete reporting or non-reporting of outcomes related to their level of significance, which have been termed “outcome reporting bias”, may influence the results of the quantitative synthesis. Lastly, though there are two different types of operative procedures (THA and TKA) summarized and analyzed in our study, the principle of fixing joint prosthesis, the implant-cement-bone interface and the antibiotic-cement-filling agent system are similar in the procedure of THA or TKA so that we could pile up two arthroplasties together through establishing suitable subgroups.

With the accelerated process of aging in our globe, more and more elderly patients who are in venerable age or have a poor physical condition desire to improve their joint function and life quality through primary TJA. And Kurtz et al. [41,42] have shown that the incidence of deep infection after primary TKA is rising and projected to reach 6.8% by 2030. Undoubtedly, it will become the focus of our joint surgeon’s attention for how to reduce the prevalence of deep prosthetic infection, a post-operative disastrous complication. Without full-length side effects of SA, the AIBC, a local drug-delivery vehicle, maybe provides a glimmer of hope for solving this world-class problem. 

## Conclusions

Compared with the PBC or SA treatments, the use of AIBC effectively reduces the deep-wound infection rate for the patients who have undergone primary total hip or knee arthroplasty, but it seems that the AIBC could not offer help to lower the superficial infection rate. In addition, the aseptic loosening rate and postoperative joint function of the AIBC group are not significantly different from the control group. Therefore, we would like to emphasize that the main benefit of AIBC is the ability to prevent deep infection without compromising patient safety in primary TJA. In the future, more larger and well-conducted RCTs on antibiotic bone cement are required to evaluate its influence on long-and short-term clinical outcomes following primary TJA.

## Supporting Information

File S1 Supporting tables.
**Table** **S1**, Characteristics of the randomized controlled trials (RCTs) included in the best evidence synthesis. Table **S2**, Jadad scale (1-5scores) for the quality evaluation of included randomized controlled trials (RCTs). Table **S3**, Post-operative superficial and deep infection rate of related randomized controlled trials (RCTs). Table **S4**, The incidence of post-operative aseptic loosening. Table **S5**, The specific description for post-operative migration. Table **S6**, Clinical joint score for hip and knee in preoperative and postoperative follow-up level.
(DOCX)

Checklist S1 **PRISMA 2009 Checklist in this meta-analysis.**
(DOC)
