# Correction: A Systematic Review and Meta-Analysis of Antibiotic-Impregnated Bone Cement Use in Primary Total Hip or Knee Arthroplasty

**DOI:** 10.1371/journal.pone.0100482

**Published:** 2014-06-11

**Authors:** The PLOS ONE Staff

The authors of this article wish to deeply apologize for the use of text verbatim from previous publications and for the lack of appropriate citation and quotations. The overlap in the text with that from previous publications relates to fragments in the text of the Introduction, Materials and Methods, and Discussion section of the article.

This paragraph under the Materials and Methods overlaps with text from the article: Yang B, Li H, Wang D, He X, Zhang C, Yang P. Systematic review and meta-analysis of perioperative intravenous tranexamic acid use in spinal surgery. PLoS One. 2013;8(2):e55436.

‘A meta-analysis and systematic review was conducted according to predefined guidelines provided by the Cochrane Collaboration (2008) [13]. All data were reported according to the Quality of Reporting for Meta-analyses provided by the Handbook for Systematic Reviews of Interventions Version 5.0.0 [14]. ’

‘If any heterogeneity was observed, the cause of heterogeneity was first analyzed and then subjected to subgroup treatment.’

The text under the Statistical Analysis section below overlaps with text from Haien Z, Yong J, Baoan M, Mingjun G, Qingyu F. Post-operative auto-transfusion in total hip or knee arthroplasty: a meta-analysis of randomized controlled trials. PLoS One. 2013;8(1): e55073.

‘The data were pooled using REVMAN 5.0 software (The Nordic Cochrane Centre, Copenhagen, Denmark). For each study, we calculated RRs with 95% confidence intervals (CIs) for dichotomous data and mean differences (MDs) with 95% CIs for continuous data. Where appropriate, we pooled the results of comparable groups of trials using the fixed-effect (Mantel-Haenszel test) or random-effect (DerSimonian-Laird method) models’.

‘ A random-effect model was used when significant heterogeneity was detected between studies (P<0.10; I2>50%). Otherwise, a fixed-effect model was used.’

The following fragments in the Introduction and Discussion section overlap with text from Parvizi et al. cited as reference 12 (Parvizi et al. Efficacy of antibiotic-impregnated cement in total hip replacement. Acta Orthop. 2008 Jun; 79(3):335-41.)

‘deep infection. A secondary aim was to evaluate whether impregnating cement with antibiotic had adverse effects on the survivorship of primary TJA’

‘addition of antibiotics to polymethylmethacrylate cement with demonstrable elution over a period of time has been shown to be effective in the treatment of established periprosthetic ‘

The following sentence in the Discussion overlaps with text by Gandhi et al. Antibiotic bone cement and the incidence of deep infection after total knee arthroplasty. J Arthroplasty. 2009 Oct;24(7):1015-8. doi: 10.1016/j.arth.2008.08.004.

‘Kurtz et al. have shown that the incidence of deep infection after primary TKA is rising and projected to reach 6.8% by 2030.’

The authors declare that the text overlap has no effect on the results and conclusions of the study and apologize for the instances of plagiarism above.
