# Supplementary material for: A Systematic Review and Meta-Analysis of Antibiotic-Impregnated Bone Cement Use in Primary Total Hip or Knee Arthroplasty
Source: PLoS One. 2013 Dec 12;8(12):e82745. doi: 10.1371/journal.pone.0082745 (PMC3861452; doi:10.1371/journal.pone.0082745)
Supplement: File S1 — Supporting tables. Table S1, Characteristics of the randomized controlled trials (RCTs) included in the best evidence synthesis. Table S2, Jadad scale (1-5scores) for the quality evaluation of included randomized controlled trials (RCTs). Table S3, Post-operative superficial and deep infection rate of related randomized controlled trials (RCTs). Table S4, The incidence of post-operative aseptic loosening. Table S5, The specific description for post-operative migration. Table S6, Clinical joint score for hip and knee in preoperative and postoperative follow-up level. (DOCX) [file pone.0082745.s001.docx]

**Supporting Information Tables**

**TableS1**. Characteristics of the RCTs included in the best evidence synthesis

| Trial | Joint (cases) | Age | Cement type | Antibiotic in cement |  | Outcomes | |
| --- | --- | --- | --- | --- | --- | --- | --- |
|  | AC:C | Mean (y) |  | (per 40g) | Infection | Radiograph | Clinical Joint Score |
| **Hip** |  |  |  |  |  |  |  |
| Pfarr 1979 | 100:100 | 65 | Palacos | gentamicin | Y | Y | N |
| Wannske 1979 | 274:202 | 63.7 | Palacos | gentamicin | Y | N | N |
| Josefsson 1981 | 821:812 | M 68 F 70 | Palacos | 0.5g gentamicin | Y | Y | N |
| Bohm 2012 | 14:11 | ≥60 | Simplex P | tobramycin | N | Y | Y |
| **Knee** |  |  |  |  |  |  |  |
| Chiu 2002 | 178:162 | AC:70 C:68 | Simplex P | 2g cefuroxime | Y | Y | Y |
| Hinarejos 2013 | 1483:1465 | 75 | Simplex P | 0.5g ERY and colistin | Y | N | N |
| **Hip and Knee** |  |  |  |  |  |  |  |
| McQueen1987 | 146:149 | 68 | CMW | 1.5g cefuroxime | Y | N | N |
| McQueen1990 | 201:200 | 67 | CMW | 1.5g cefuroxime | Y | N | N |

RCTs=randomized controlled trials; AC=antibiotic-cement group; C=control group; ERY=erythromycin; Y=yes; N=no; M=male; F=female

**Table S2**. Jadad scale (1-5 scores) for the quality evaluation of included RCTs

| Inclusion study | Design | Follow-up (months) | Random | Concealment | Blinding | Drop-out( cases) | Jadad Score |
| --- | --- | --- | --- | --- | --- | --- | --- |
| **Hip** |  |  |  |  |  |  |  |
| Pfarr 1979 | RCT | 24 | Y | UC | N | N | 1 |
| Wannske 1979 | RCT | 29 | Y | UC | N | N | 1 |
| Josefsson 1981 | RCT | 24 | Y | UC | N | Y(52) | 2 |
| Bohm 2012 | RCT | 24 | Y | Y | Y (triple-blind) | Y(10) | 4 |
| **Knee** |  |  |  |  |  |  |  |
| Chiu 2002 | RCT | 49 | Y | UC | N | N | 1 |
| Hinarejos 2013 | RCT | 12 | Y | Y | N | Y(52) | 3 |
| **Hip and Knee** |  |  |  |  |  |  |  |
| McQueen1987 | RCT | 3 | Y | UC | N | Y | 2 |
| McQueen1990 | RCT | 24 | Y | UC | Y (single-blind) | Y(4) | 2 |

RCTs=randomized controlled trials; Y=yes ; N=no; UC=unclear

**Table S3**. Post-operative superficial and deep infection rate of related RCTs (cases and percents)

| Studies | Superficial Infection | | Deep Infection | |
| --- | --- | --- | --- | --- |
|  | AC C | | AC C | |
| **Hip** |  |  |  |  |
| Pfarr 1979 | UC | UC | 0(0%) | 0(0%) |
| Wannske 1979 | UC | UC | 3(1.1%) | 12(5.9%) |
| Josefsson 1981 | 71(8.6%) | 49(6.0%) | 3(0.4%) | 13(1.6%) |
| **Knee** |  |  |  |  |
| Chiu 2002 | 2(1.1%) | 2(1.2%) | 0(0%) | 5(3.1%) |
| Hinarejos 2013 | 27(1.82%) | 18(1.23%) | 20(1.35%) | 20(1.37%) |
| **Hip and Knee** |  |  |  |  |
| McQueen1987 | 11(7.5%) | 10(6.7%) | 1(0.7%) | 2(1.3%) |
| McQueen1990 | 18(9%) | 8(4%) | 2(1.0%) | 2(1.0%) |

UC=unclear; AC=antibiotic-cement group; C=control group

**Table S4.** The incidence of post-operative aseptic loosening (cases and percents)

| Studies | AC | C | ***P*** value |
| --- | --- | --- | --- |
| **Hip** |  |  |  |
| Pfarr 1979 | 18(18%) | 8(8%) | UC |
| Josefsson 1981 | 13(1.6%) | 27(3.3%) | 0.03 |
| **Knee** |  |  |  |
| Chiu 2002 | 1(0.6%) | 0(0%) | NS* |

* No statistical differences ; UC=unclear; AC=antibiotic-cement group; C=control group

**Table S5.** The specific description for post-operative migration

| Studies | Translation (mm) Rotation (degrees) | | | | | |
| --- | --- | --- | --- | --- | --- | --- |
|  | AC | C | ***P*** value | AC | C | ***P*** value |
| **Hip** |  |  |  |  |  |  |
| Bohm Eric 2012 |  |  |  |  |  |  |
| Transversal | 0.01±0.13 | -0.01±0.09 | 0.8 | -0.11±0.17 | 0.01±0.20 | 0.1 |
| Longitudinal | -0.77±0.36 | -0.71±0.30 | 0.6 | 0.84±0.93 | 0.60±0.73 | 0.5 |
| Sagittal | -0.17±0.23 | -0.09±0.21 | 0.4 | -0.04±0.12 | 0.00±0.11 | 0.4 |

AC=antibiotic-cement group; C=control group

**Table S6.** Clinical joint score for hip and knee in preoperative and postoperative follow-up level

| Studies | Preoperative Postoperative | | | | | |
| --- | --- | --- | --- | --- | --- | --- |
|  | AC | C | ***P*** value | AC | C | ***P*** value |
| **Hip** |  |  |  |  |  |  |
| Bohm 2012**^a^** | 47(30–80) | 50(22–70) | 0.7 | 86(58–100) | 88(67–100) | 0.8 |
| **Knee** |  |  |  |  |  |  |
| Chiu 2002**^b^** | 45±7.6 | 48±7.7 | 0.431 | 90±3.8 | 88±8.2 | 0.523 |

1. the use of The Harris Hip Score; b. the use of The Hospital for Special Surgery Score

AC=antibiotic-cement group; C=control group
