# Supplementary material for: A Systematic Review and Meta-Analysis of Antibiotic-Impregnated Bone Cement Use in Primary Total Hip or Knee Arthroplasty
Source: PLoS One. 2013 Dec 12;8(12):e82745. doi: 10.1371/journal.pone.0082745 (PMC3861452; doi:10.1371/journal.pone.0082745)
Supplement: Checklist S1 — PRISMA 2009 Checklist in this meta-analysis. (DOC) [file pone.0082745.s002.doc]

| **Section/topic** | **#** | **Checklist item** | **Reported on page #** |
| --- | --- | --- | --- |
| **TITLE** | | |  |
| Title | 1 | A Systematic Review and Meta-Analysis of Antibiotic-Impregnated Bone Cement Use in Primary Total Hip or Knee Arthroplasty | Title |
| **ABSTRACT** | | |  |
| Structured summary | 2 | Background: Antibiotic-impregnated bone cement (AIBC) has been widely used for the treatment of infected revision arthroplasty, but its [routine](app:ds:prophylactic) use in primary total joint arthroplasty (TJA) remains considerably controversial. With this meta-analysis of published randomized controlled trials, we intended to assess the antimicrobial efficacy and safety of AIBC for its [prophylactic](app:ds:prophylactic) use in primary TJA.  Objectives: To evaluate the efficacy of AIBC, we conducted a Cochrane systematic review that combined all available data from randomized controlled trials(RCTs).  Data sources: Studies published in Medline, Embase, CBMdisc and the Cochrane Library until June, 2013.  Study eligibility criteria, participants, and interventions: patients undergoing primary total knee or hip arthroplasty; types of antibiotic administered in the trial group was AIBC and the control was plain bone cement or systemic antibiotic, irrespective of dose and route of administration. Patients were excluded from the trials if they had received any revision arthroplasty or primary study patients had a poor physical condition, such as diabetes, malignant tumor.  Study appraisal and synthesis methods: The data were pooled using REVMAN 5.0 software (The Nordic Cochrane Centre, Copenhagen, Denmark). For each study, we calculated relative risks (RRs) with 95% confidence intervals (CIs) for dichotomous data and Mean Differences (MDs) with 95% CIs for continuous data.  Results: We found that the prophylactic use of AIBC could lower the deep infection rate in primary TJA, while AIBC did not show an improvement in reducing the superficial infection rate compared with the control.  Conclusions and implications of key findings: To our knowledge, this is an updated meta-analysis to compare the clinical results between AIBC and the control, in primary joint replacement patients. | Abstract |
| **INTRODUCTION** | | |  |
| Rationale | 3 | Postoperative infection following primary total joint arthroplasty (TJA), especially deep-wound infection, is still a serious complication. Compared with systemic antibiotics which cannot provide sufficient antibiotic concentrations and may cause systemic toxicity, antibiotic-impregnated bone cement (AIBC) is now regarded as an effective method to prevent or treat deep infection following TJA. But there have also been some worries regarding the addition of antibiotics to bone cement and its routine use in primary TJA. The main disadvantages are the possible development of antibiotic resistance, allergic reaction, toxicity, and possible compromise of the mechanical properties of bone cement, and increased cost. | Objectives |
| Objectives | 4 | The main objective of this study was to compare the antimicrobial efficacy and safety of antibiotic-impregnated bone cement (AIBC) for its [prophylactic](app:ds:prophylactic) use in primary TJA. | Objectives |
| **METHODS** | | |  |
| Protocol and registration | 5 | The data were extracted by 2 reviewers independently to ensure accuracy. In cases of disagreement, consensus was reached by discussion and was eventually determined by the senior author. Study quality was evaluated according to the five-point Jadad scale.  No registration information and registration number. | Inclusion and Exclusion Criteria |
| Eligibility criteria | 6 | Study characteristics: the numbers of patients following primary total joint arthroplasty ; types of antibiotic administered, antibiotic-impregnated bone cement in the trial group and plain bone cement or systemic antibiotic in the control; post-operative infection rate; post-operative radiographic evaluation (the aseptic loosening) ;clinical joint score.  The published results of relevant trials until June 2013 without language limitation. | Inclusion and Exclusion Criteria |
| Information sources | 7 | We searched for the published results of relevant trials in Medline, Embase, CBMdisc and the Cochrane Library. When necessary, the authors of the articles were contacted for original information.  Date last searched: June 2013. | Literature Search |
| Search | 8 | The key words used were “hip arthroplasty/ replacement”, “knee arthroplasty/replacement”, “joint arthroplasty/replacement”, “antibiotic cement”, “cement”, and “randomized controlled trial”. In order to collect relevant literature as many as possible, we also used “bone cement”, “antibiotic”, “gentamicin”, “cefuroxime”, “tobramycin”, and “acrylic” as the primary search terms as well as combined with various limiting requirements such as “arthroplasty”, “hip replacement”, and “knee replacement”.  Search strategy: MEDLINE (For Example)   1. (cement[Title/Abstract]) OR antibiotic cement[Title/Abstract] 2. "Bone Cements"[Mesh] 3. 1 or 2 4. (joint replacement[Title/Abstract]) OR joint arthroplasty[Title/Abstract] 5. "Arthroplasty, Replacement"[Mesh] 6. 4 or 5 7. (hip replacement[Title/Abstract]) OR hip arthroplasty[Title/Abstract] 8. "Arthroplasty, Replacement, Hip"[Mesh] 9. 7 or 8 10. (knee replacement[Title/Abstract]) OR knee arthroplasty[Title/Abstract] 11. "Arthroplasty, Replacement, Knee"[Mesh] 12. 10 or 11 13. randomized[Title/Abstract] 14. ("Randomized Controlled Trial" [Publication Type]) OR "Randomized Controlled Trials as Topic"[Mesh] 15. 13 or 14 16. 3 and 6 and 15 17. 3 and 9 and 15 18. 3 and 12 and 15 | Literature Search |
| Study selection | 9 | First, records after duplicates removed by Endnote software, then screening the 273 records included;  Second, browsing the titles and abstracts of these articles, excluding 242 articles;  Third, records screened for detailed information, excluding 17 articles with reasons: 1, not RCTs. 2, not the primary joint arthroplasty. Then there was 14 full-text articles assessed for further screening.  Last, 8 studies included in quantitative synthesis for meta-analysis. | See Flow Diagram |
| Data collection process | 10 | The data were extracted by 2 reviewers independently to ensure accuracy. In cases of disagreement, consensus was reached by discussion of all investigators and was eventually determined by the senior author. | Inclusion and Exclusion Criteria |
| Data items | 11 | Participants: patients undergoing primary total knee or hip arthroplasty, without considering revision of the replacement.  Interventions: bone cement and antibiotic  Comparisons: the trial, antibiotic-impregnated bone cement(AIBC) and the control, plain bone cement or systemic antibiotic  Outcomes: The primary outcome assessed in this analysis was postoperative infection rate between the AIBC and control group, which contained superficial and deep infection rate. The secondary outcome measures included radiographic evaluation (the aseptic loosening) and clinical joint score. | Outcome Measures  and Table S1 |
| Risk of bias in individual studies | 12 | To ascertain the validity of eligible randomized trials, pairs of reviewers working independently and with adequate reliability determined the adequacy of randomization and concealment of allocation, blinding of patients, health care providers, data collectors, and outcome assessors | Methodological Quality Assessment  and Table S2 |
| Summary measures | 13 | The principal summary measures: For each study, we calculated relative risks (RRs) with 95% confidence intervals (CIs) for dichotomous data and Mean Differences (MDs) with 95% CIs for continuous data. | Statistical Analysis |
| Synthesis of results | 14 | we pooled the results of comparable groups of trials using both the fixed effects (Mantel-Haenszel test) and random effects (DerSimonian-Laird method) models. A random effects model was used when significant heterogeneity was detected between studies (P<.10; I^2^>50%). | Statistical Analysis |

Page 1 of 2

| **Section/topic** | **#** | **Checklist item** | **Reported on page #** |
| --- | --- | --- | --- |
| Risk of bias across studies | 15 | For each trial we plotted the effect by the inverse of its standard error. The symmetry of such ‘funnel plots’ was assessed both visually and formally with Egger’s test, to see if the effect decreased with increasing sample size. | Statistical Analysis |
| Additional analyses | 16 | Considering whether bone cement was used in the control group, the surgical wound infection (superficial and deep) rate data were divided into 2 subgroups (AIBC vs. PBC and AIBC vs. SA) in each study. If any heterogeneity was observed, the cause of heterogeneity was first analyzed and then subjected to subgroup treatment. | Subgroup Analysis and Investigation of Heterogeneity |
| **RESULTS** | | |  |
| Study selection | 17 | 273 studies screened, 14 full-text articles assessed for eligibility, and 8 articles assessed included in the meta-analysis. Reasons for exclusions at each stage, seen in the flow diagram. | See Flow Diagram |
| Study characteristics | 18 | All 8studies finally selected for the analysis were randomised controlled trials published in English and German.  In total, 6318 arthroplasties were included in our study; 3217 of these arthroplasties received antibiotic-impregnated bone cement and 3101 arthroplasties served as the control. | Literature Search  and Table S1 |
| Risk of bias within studies | 19 | Present data on risk of bias of each study and, if available, any outcome level assessment (see item 12). | Methodological Quality Assessment |
| Results of individual studies | 20 | postoperative superficial and deep infection: See forest plots in Figure 2-5 and Table S3.  radiographic assessment: See Table S4 and S5.  clinical joint score: See Table S6. | Figure 2-5  Table S3-S6 |
| Synthesis of results | 21 | 1. postoperative superficial infection:( RRs, 1.47; 95% CIs, 1.13 to 1.91; P= 0.004)  2. postoperative deep infection:( RRs, 0.41; 95% CIs, 0.17 to 0.97; P= 0.04)  3. postoperative deep infection: Gentamicin (RRs, 0.21; 95% CIs, 0.08 to 0.50; P=0.0005) and Cefuroxime (RRs, 0.36; 95% CIs, 0.11 to 1.20; P= 0.10) | Post-operative Superficial and Deep Infection Rate |
| Risk of bias across studies | 22 | To explore the heterogeneity, a funnel plot was drawn. The funnel plot shows evidence of considerable asymmetry. | Publication bias |
| Additional analysis | 23 | Through the sensitivity analysis, we found that two studies from Josefsson et al. and [Hinarejos](http://www.ncbi.nlm.nih.gov/pubmed?term=Hinarejos P%5BAuthor%5D&cauthor=true&cauthor_uid=23636182)et al. reported different research results on deep infection rate and the data determined the final analysis results in their respective subgroups. However, with or without these two studies, our overall pooled results both revealed that the trial group had a better effect than the control group in the prevention of deep infection (P<0.05). | Discussion |
| **DISCUSSION** | | |  |
| Summary of evidence | 24 | For deep infection intervention, compared with PBC and SA, our comparative analysis had demonstrated a clear benefit of AIBC which led to a statistically significant reduction in the incidence of deep infection following primary TJA. And bone cement containing gentamicin has a better effect in preventing deep infection than the cefuroxime-loaded cement.  For superficial infection prevention, our results hinted that when SA was used as the reference, the incidence of superficial wound infection was higher in the AIBC group than that in the control group.  There is no significant difference on mechanical properties of bone cement, antibiotic resistance, allergic reaction, toxicity, and increased cost between AIBC and the control. | Discussion |
| Limitations | 25 | The main limitation of this meta-analysis is that there is a paucity of eligible randomized controlled trials and the original studies are conducted in different hospitals around the world with different patient populations. Publication bias might account for some of the effects we observed. Smaller trials are analyzed with less methodological rigor than larger studies, and an asymmetrical funnel plot suggests that selective reporting may have led to an overestimation of effect sizes in small trials.  More larger and well-conducted RCTs on antibiotic bone cement are required to evaluate its influence on long-and short-term clinical outcomes following primary TJA. | Limitations |
| Conclusions | 26 | Compared with the PBC or SA treatments, the use of AIBC effectively reduces the deep-wound infection rate for the patients who have undergone primary total hip or knee arthroplasty, but it seems that the AIBC could not offer help to lower the superficial infection rate. In addition, the aseptic loosening rate and postoperative joint function of the AIBC group are not significantly different from the control group. Therefore, we would like to emphasize that the main benefit of AIBC is the ability to prevent deep infection without compromising patient safety in primary TJA. | Conclusions |
| **FUNDING** | | |  |
| Funding | 27 | No current external funding sources for this study. | None |

*From:*  Moher D, Liberati A, Tetzlaff J, Altman DG, The PRISMA Group (2009). Preferred Reporting Items for Systematic Reviews and Meta-Analyses: The PRISMA Statement. PLoS Med 6(6): e1000097. doi:10.1371/journal.pmed1000097

For more information, visit: **www.prisma-statement.org**.

Page 2 of 2
